# Correlation between Metabolic Parameters and Warfarin Dose in Patients with Heart Valve Replacement of Different Genotypes

**DOI:** 10.31083/j.rcm2504128

**Published:** 2024-04-01

**Authors:** Xiaowu Wang, Diancai Zhao, Jipeng Ma, Xia Wang, Jincheng Liu

**Affiliations:** ^1^Department of Cardiovascular Surgery, Xijing Hospital, Fourth Military Medical University, 710032 Xi'an, Shaanxi, China; ^2^Department of Health Statistics, Faculty of Preventive Medicine, Fourth Military Medical University, 710032 Xi'an, Shaanxi, China

**Keywords:** warfarin, pharmacogenetics, liver function, kidney function, metabolic index, dosing algorithm

## Abstract

**Background:**

Warfarin has become the first choice for anticoagulation in patients who need lifelong anticoagulation due to its clinical efficacy and low price. However, the anticoagulant effect of warfarin is affected by many drugs, foods, etc. accompanied by a high risk of bleeding and embolism. The Vitamin K epoxide reductase complex 1 (*VKORC1*) and Cytochrome P450 2C9 (*CYP2C9*) genotypic variation can influence the therapeutic dose of warfarin. However, it is not clear whether there is a correlation between warfarin dose and liver function, kidney function and metabolic markers such as uric acid (UA) in patients with different genotypes. We performed a single-center retrospective cohort study to evaluate the factors affecting warfarin dose and to establish a dose conversion model for warfarin patients undergoing heart valve replacement.

**Methods:**

We studied 343 patients with a mechanical heart valve replacement, compared the doses of warfarin in patients with different warfarin-related genotypes (*CYP2C9* and *VKORC1*), and analyzed the correlation between liver function, kidney function, UA and other metabolic markers and warfarin dose in patients with different genotypes following heart valve replacement.

**Results:**

Genotype analysis showed that 72.01% of patients had CYP2C9*1/*1 and *VKORC1* mutant AA genotypes. Univariate regression analysis revealed that the warfarin maintenance dose was significantly correlated with gender, age, body surface area (BSA), UA and genotype. There was no correlation with liver or kidney function. Multiple linear regression analysis showed that BSA, genotype and UA were the independent factors influencing warfarin dose.

**Conclusions:**

There is a significant correlation between UA content and warfarin dose in patients with heart valve replacement genotypes CYP2C9*1/*1/VKORC1(GA+GG), CYP2C9*1/*1/VKORC1AA and CYP2C9*1/*1/VKORC1AA.

## 1. Introduction

Warfarin anticoagulation has a narrow therapeutic range. The drug metabolism and efficacy of warfarin are affected by genetics, age, diet, and weight [1, 2, 3]. Therefore, the dose of warfarin needs to be continuously adjusted. In recent years, direct oral anticoagulants (DOACs) have replaced warfarin to some extent, but warfarin is still preferred in many patients with heart valve replacement who require lifelong anticoagulation due to its better anticoagulant effect and lower price [4, 5]. Therefore, it is of great clinical value to clarify the factors affecting the drug metabolism and efficacy of warfarin in patients undergoing heart valve replacement to be able to develop a model to calculate the warfarin dose in patients to more rapidly achieve therapeutic levels which can reduce bleeding, thrombosis and other complications caused by inappropriate dosage.

Different genotypes of Vitamin K epoxide reductase complex 1 (*VKORC1*) and Cytochrome P450 2C9 (*CYP2C9*) can affect the dose of warfarin [6]. The therapeutic dose of warfarin can be estimated by genotyping single nucleotide polymorphisms (SNPs) of patients that affect warfarin metabolism or sensitivity. Pharmacogenetic-based therapy can improve the safety of anticoagulant therapy by providing a model to assess the therapeutic doses necessary for achieving anticoagulation with warfarin. In patients with abnormal metabolism manifested by liver and kidney dysfunction and uric acid (UA), the metabolism, distribution, absorption and excretion of warfarin is altered. Therefore, there is an urgent need to elucidate the correlation between the dose of warfarin and metabolic markers in patients with different genotypes.

In this study, we aimed to clarify the correlation between the warfarin dose and metabolic indices in patients undergoing heart valve replacement with different warfarin-related genotypes, to further clarify which indicators are independent factors affecting warfarin dose, and establish a calculation model for warfarin dosages, so as to provide a theoretical basis for shortening the time to achieve therapeutic anticoagulation and to minimize complications associated with warfarin therapy.

## 2. Materials and Methods

### 2.1 Study Population and Design

This study is a single-center retrospective study with 343 participants from patients who underwent mechanical heart valve replacement at the Department of Cardiovascular Surgery of Xijing Hospital of Fourth Military Medical University from January 1, 2021 to August 31, 2022. Inclusion Criteria included: (1) Data collected three months after heart valve replacement; (2) Oral warfarin anticoagulation supervised by a physician; (3) Patients who were 16–65 years old; (4) Patients who were able to provide informed consent. If the patient was unable to sign due to aphasia or motor dysfunction but agrees to participate, the signature was signed by a family member. Exclusion Criteria included: (1) Patients who did undergo mechanical heart valve replacement but required oral warfarin or other diseases; (2) Patients were younger than 16 years old or older than 65 years old; (3) Patients with serious warfarin metabolic diseases, including severe gastrointestinal dysfunction or malignant tumors; (4) Patients who had congenital coagulation dysfunction; (5) Patients in whom oral warfarin were interrupted for more than 3 consecutive days within 3 months following surgery.

### 2.2 Patient Demographics

Baseline patient information included age, gender, ethnicity, height, weight, and body surface area (BSA). Clinical data included the type of valve replacement, PT-INR (prothrombin time-international normalized ratio), dose of warfarin; warfarin genotyping (*CYP2C9* and *VKORC1*), and adverse events. Metabolic indicators included liver function, assessed by alanine aminotransferase (ALT), aspartate aminotransferase (AST), total bilirubin (TBIL), total protein (TP), and albumin (ALB) and kidney function manifested by blood urea nitrogen (BUN), and creatinine (Cr). UA and total calcium (TCa) content were also measured.

### 2.3 Genetic Subtyping Genotype

The laboratory method of genetic testing is described in more detail in the manuscript: After the informed consent was signed, peripheral blood was collected and genomic DNA was extracted for genotyping. DNA was extracted from blood leukocytes using nucleic acid extraction and a purification reagent produced by Shanghai Bairo Co., Ltd. In order to obtain polymorphism of CYP2C9 gene CYP2C9*3 (C.1075A >C) and VKORC1 (C.-1639G >A), primers designed and synthesized in advance were used for polymerase chain reaction (PCR) amplification in the nearby region. The primers at the CYP2C9*3 site were as follows: forward primer, $5^{\prime }$-ACGTGTGATTGGCAGAAACC-$3^{\prime }$; Reverse primer, $5^{\prime }$-GCCAGACACTAGGACCTGTT-$3^{\prime }$; The primer pairs at VKORC1 (C.-1639G >A) site were: forward primer, $5^{\prime }$-CTCCCGGCATTATCCCATCT-$3^{\prime }$, reverse primer, $5^{\prime }$-ACGCCAGAGGAAGAGAGTTC-$3^{\prime }$, and the product was sequenced after gel purification. The sequences were compared with the reference sequences CYP2C9 (NM_000771.4) and VKORC1 (NM_206824.3), respectively, to verify the presence of mutations in patient samples. CYP2C9*1 and CYP2C9*3, as well as VKORC1 mutant AA, VKORC1 mutant GA, and VKORC1 mutant GG, were tested in blood samples for CYP2C9*1 and CYP2C9*3 deficiency in Asian populations [7]. Single nucleotide polymorphisms were detected by TaqMan PCR (Taicang, Beijing, China).

### 2.4 Statistical Analysis

Values are expressed as means ± standard deviations or as percentages. Numerical data was described using composition ratios, and the chi square test was used for comparison between the groups. Multiple linearregression analysis was used for multifactorial analysis. SPSS 24.0 software (Windows version 11; SPSS Inc., Chicago, IL, USA) was used for the statistical analysis. *p* < 0.05 was considered statistically significant.

## 3. Results

### 3.1 Patient Characteristics

We recorded the age, sex, ethnicity, height, weight, BSA, adverse events, warfarin dose, and type of heart valve replacement in all the participants. Indicators of liver function were ALT, TP, AST, ALB, ALP and gamma-glutamyltransferase (GGT). Kidney function indicators include BUN, Cr, and UA. We also tested TCa levels. (Table 1). During follow-up, three adverse events including 2 hemorrhages and 1 thrombosis were recorded. The original data in Table 1 can be found in **Supplementary Data 1**.

**Table 1.** **Patient characteristics**.

Variables		N = 343
Age (years, mean ± SD)		(48.84 ± 11.07)
	16–18 years (n, %)	2 (0.59)
	18–60 years (n, %)	305 (88.92)
	>60 years (n, %)	36 (10.49)
Male (n, %)		208 (60.64)
Ethnicity (n, %)		
	Han	339 (98.83)
	Other	4 (1.17)
Height (cm, mean ± SD)		166.46 ± 8.21
Weight (kg, mean ± SD)		65.51 ± 11.81
BSA (/$m^{2}$)		1.82 ± 0.18
Adverse event (n, %)		3 (0.87)
warfarin dose (mg, mean ± SD)		2.95 ± 0.93
Type of surgery (n, %)		
	AVR only	190 (55.39)
	MVR only	74 (21.57)
	AVR and MVR	73 (21.28)
	TVR	6 (1.76)
Liver function		
	ALT (U/L, mean ± SD)	39.69 ± 37.22
	AST (U/L, mean ± SD)	43.19 ± 29.48
	TP (g/L, mean ± SD)	65.39 ± 6.96
	ALB (g/L, mean ± SD)	35.97 ± 3.85
	ALP (U/L, mean ± SD)	80.27 ± 35.23
	GGT (U/L, mean ± SD)	65.89 ± 57.87
Kidney function		
	BUN (mmol/L, mean ± SD)	8.20 ± 5.61
	Cr (µmol/L, mean ± SD)	73.30 ± 33.98
	UA (µmol/L, mean ± SD)	323.30 ± 88.06
	TCa (mmol/L, mean ± SD)	2.31 ± 0.15

Data are presented as mean (SD) or absolute (percentage) values. Abbreviations: SD, standard deviation; BSA, body surface area; AVR, aortic valve replacement; MVR, mitral valve replacement; TVR, tricuspid valve replacement; ALT, alanine aminotransferase; AST, aspartate aminotransferase; TP, total protein; ALB, albumin; ALP, alkaline phosphatase; GGT, gamma-glutamyltransferase; BUN, blood urea nitrogen; Cr, creatinine; UA, uric acid; TCa, total calcium; N, number of patients.

### 3.2 Distribution of CYP2C9 and VKORC1 Genotype and their Correlation with Warfarin Maintenance Doses

A total of 343 patients completed genotype detection and clinical index examination. Genotyping results showed that 90.38% patients had CYP2C9*1/*1, including 72.01% CYP2C9*1/*1/*VKORC1* mutant AA, 18.37% CYP2C9*1/*1/*VKORC1*(GA+GG). 9.62% patients had CYP2C9*1/*3, including 8.16% CYP2C9*1/*1/*VKORC1* mutant AA, 1.46% CYP2C9*1/*1/*VKORC1*(GA+GG). CYP2C9*3/*3 genotype was not detected (Table 2).

**Table 2.** ***CYP2C9* and *VKORC1* genotypes**.

	VKORC1 genotypes		
CYP2C9	AA	GA	GG
^*^1/^*^1	247 (72.01)	60 (17.49)	3 (0.88)
^*^1/^*^3	28 (8.16)	4 (1.17)	1 (0.29)
^*^3/^*^3	0	0	0

*CYP2C9*, Cytochrome P450 2C9; *VKORC1*, Vitamin K epoxide reductase complex 1.

**Fig. 1.**
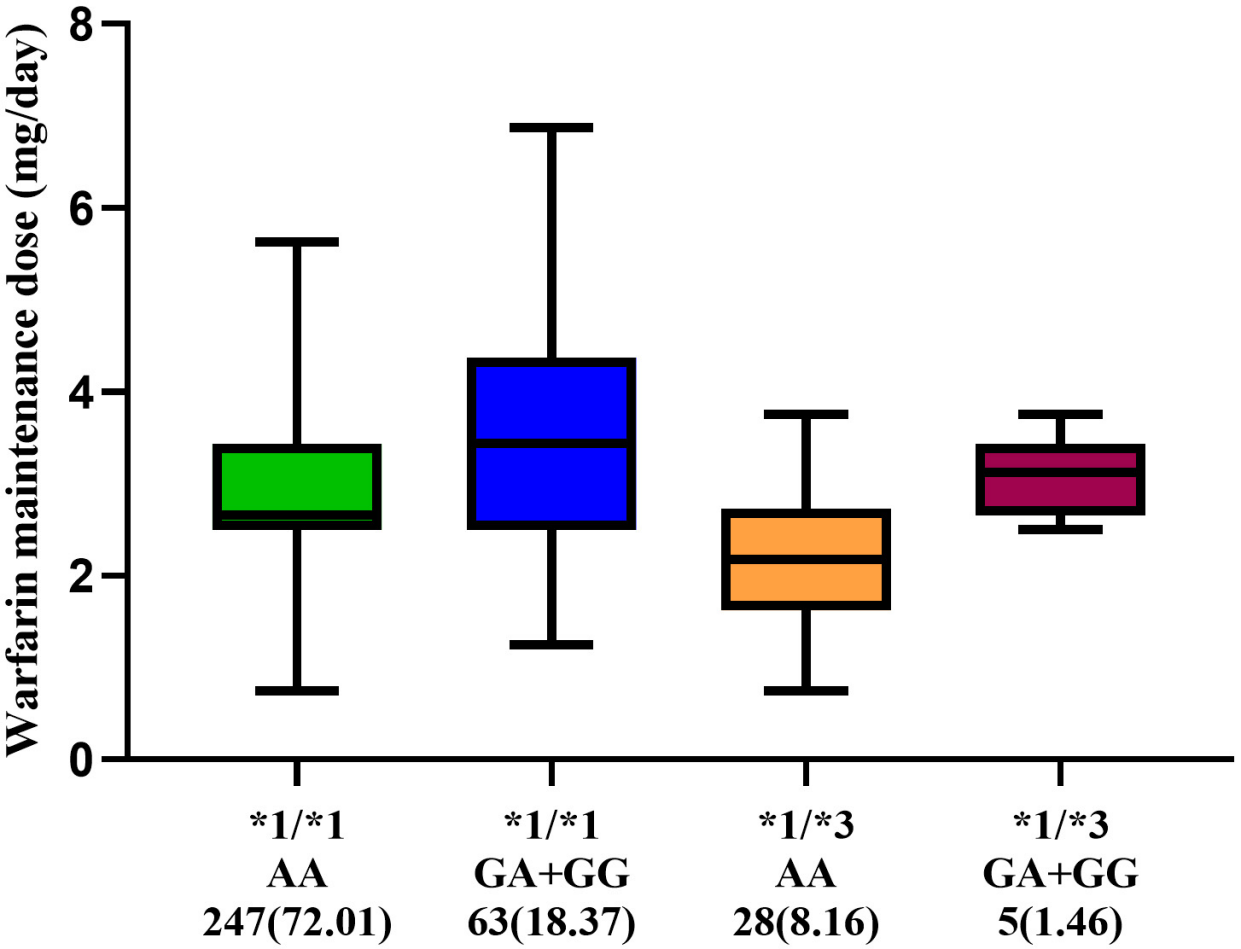
**Dosage of Warfarin in patients with different genotypes**. The central horizontal bar in the figure represents the median, and the lower and upper limits represent the 25th and 75th percentiles, respectively. The original data in Table 2 can be found in **Supplementary Data 1**.

The target international normalized ratio (INR) value of warfarin anticoagulation in Asian patients is low [8]. As a result of our clinical anticoagulation treatment, the INR value of warfarin anticoagulation in patients with heart valve replacement was maintained between 1.5 and 2.5, which was safe and resulted in good clinical efficacy. The maintenance dose of warfarin in patients with different genotypes *VKORC1* and *CYP2C9* is shown in Fig. 1. The dose of CYP2C9*1/*1/*VKORC1* mutant AA group (n = 247) was 2.90 ± 0.79 mg/day while the dose was the highest in the CYP2C9*1/*1/*VKORC1*(GA+GG) group (n = 63) at 3.50 ± 1.20 mg/day. The dose in the CYP2C9*1/*3/*VKORC1* mutant AA group (n = 28) was the lowest at 2.3 ± 0.84 mg/day, and the dose in the CYP2C9*1/*3/*VKORC1*(GA+GG) group (n = 5) was 3.10 ± 0.46 mg/day. The original data in Fig. 1 can be found in **Supplementary Data 1**.

Therefore, genotype frequencies for the CYP2C9 and VKORC1 genes were divided into four groups.

### 3.3 Univariate Linear Regression Analysis of the Correlation between Warfarin Dosage and Clinical Indicators

Continuous variables were analyzed with bivariate linear correlations (age, BSA, liver function, kidney function, and total calcium). Univariate grouping variables were divided into two groups with *t*-test analysis (sex, ethnicity) and divided into more than two groups by analysis of variance (genotype) (Table 3). The original data in Table 3 can be found in **Supplementary Data 2**.

**Table 3.** **Univariate association between variables and warfarin dose**.

Variables	r/t/F	*p* value
Sex	2.040	${\mathrm{0.042}}^{*}$
Age	–0.154	<0.004^**^
BSA (/$m^{2}$)	0.171	${\mathrm{0.02}}^{*}$
Ethnicity	0.472	0.637
ALT (U/L)	0.057	0.289
AST (U/L)	–0.020	0.708
TBIL (µmol/L)	–0.071	0.187
TP (g/L)	–0.064	0.239
ALB (g/L)	–0.011	0.835
ALP (U/L)	–0.073	0.176
GGT (U/L)	–0.014	0.800
BUN (mmol/L)	–0.027	0.612
Cr (µmol/L)	0.068	0.210
UA (µmol/L)	–0.121	${\mathrm{0.025}}^{*}$
TCa (mmol/L)	0.041	0.454
CV1	5.208	${\mathrm{0.014}}^{*}$
CV2	27.270	<0.001^***^
CV3	14.069	<0.001^***^
CV4	0.060	0.792

^*^p < 0.05, ^**^p < 0.01, ^***^p < 0.001. Abbreviations: BSA, body surface area; ALT, alanine aminotransferase; AST, aspartate aminotransferase; TP, total protein; ALB, albumin; ALP, alkaline phosphatase; GGT, gamma-glutamyltransferase; BUN, blood urea nitrogen; Cr, creatinine; UA, uric acid; TCa, total calcium; CV1, CYP2C9*1/*1/*VKORC1*AA; CV2, CYP2C9*1/*1/*VKORC1*(GA+GG); CV3, CYP2C9*1/*3/*VKORC1*AA; CV4, CYP2C9*1/*3/*VKORC1*(GA+GG); TBIL, total bilirubin; *CYP2C9*, Cytochrome P450 2C9; *VKORC1*, Vitamin K epoxide reductase complex 1.

The statistical results showed that gender, age, BSA, UA and genotype of the patients were significantly correlated with the maintenance dose of warfarin, while the ethnicity, liver function, kidney function and total calcium content of the patients were not significantly correlated with the maintenance dose of warfarin.

**Fig. 2.**
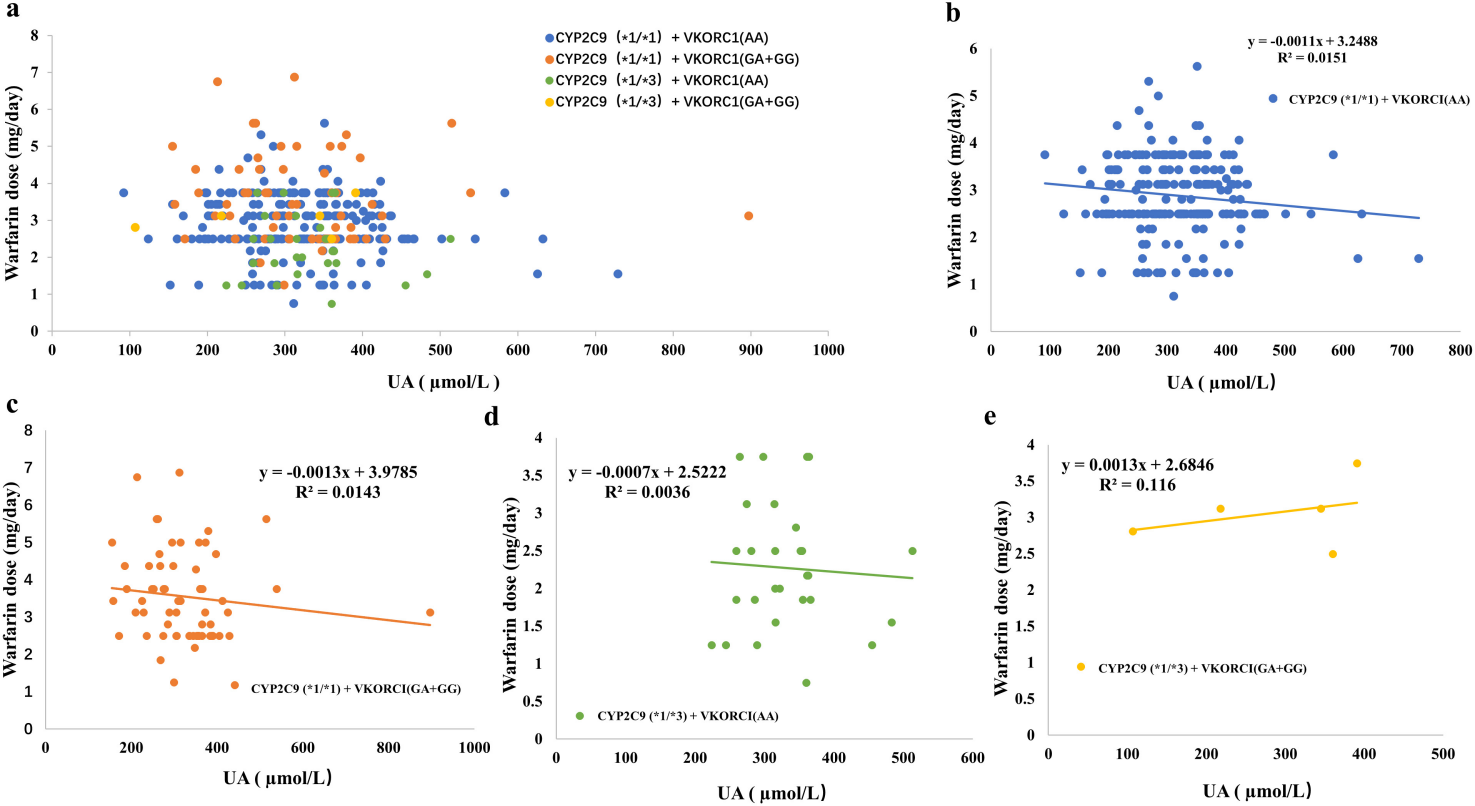
**Correlation between UA content and Warfarin dose in patients with different genotypes**. (a) Correlation between UA content and Warfarin dose in all patients. (b) Correlation between UA content and warfarin dose in patients with CYP2C9*1/*1/*VKORC1*AA genotype. (c) Correlation between UA content and warfarin dose in patients with CYP2C9*1/*1/*VKORC1*(GA+GG) genotype. (d) Correlation between UA content and warfarin dose in patients with CYP2C9*1/*3/*VKORC1*AA genotype. (e) Correlation between UA content and warfarin dose in patients with CYP2C9*1/*3/*VKORC1*(GA+GG) genotype. *CYP2C9*, Cytochrome P450 2C9; *VKORC1*, Vitamin K epoxide reductase complex 1; UA, uric acid.

The correlation between UA content and warfarin dose in patients with different genotypes was further clarified (Fig. 2a). The data revealed that the maintenance dose of warfarin in CYP2C9*1/*1/*VKORC1* mutant AA group, CYP2C9*1/*1/*VKORC1*(GA+GG) group and CYP2C9*1/*3/*VKORC1*AA group was negatively correlated with UA content (Fig. 2b–d). A high UA increased the anticoagulant effect of warfarin and reduced the maintenance dose of warfarin in these patients. The dose of warfarin in patients with CYP2C9*1/*3/*VKORC1*(GA+GG) was positively correlated with UA content (Fig. 2e). The original data in Fig. 2 can be found in **Supplementary Data 1**.

### 3.4 Multiple Linear Regression Analysis 

In univariate analysis, clinical variables that may be relevant to warfarin maintenance doses included gender, age, BSA, UA, and genotype (indicators of *p* < 0.05 in Table 3).

These indicators were selected for multiple linear regression to explore the factors that determine the dose of warfarin. Statistical results showed that the factors associated with warfarin dose included BSA, UA, and genotype (indicators of *p* < 0.05 in Table 4). The original data in Table 4 can be found in **Supplementary Data 3**.

**Table 4.** **Multiple linear regression analysis**.

Variables	B value	Standard error	*p* value
Sex	0.053	0.117	0.650
Age	–0.007	0.004	0.115
BSA (/$m^{2}$)	0.820	0.312	0.009^**^
UA (µmol/L)	–0.001	0.001	${\mathrm{0.020}}^{*}$
Genotypes			
CYP2C9*1/*1/*VKORC1*(GA+GG)	0.692	0.121	<0.001^***^
CYP2C9*1/*3/*VKORC1*AA	–0.553	0.171	0.001^**^
CYP2C9*1/*3/*VKORC1*(GA+GG)	0.163	0.387	0.673

Note: ^*^p < 0.05, ^**^p < 0.01, ^***^p < 0.001. B, regression coefficient; BSA, body surface area; UA, uric acid; CV2, CYP2C9*1/*1/*VKORC1*(GA+GG); CV3, CYP2C9*1/*3/*VKORC1*AA; CV4, CYP2C9*1/*3/*VKORC1*(GA+GG); *CYP2C9*, Cytochrome P450 2C9; *VKORC1*, Vitamin K epoxide reductase complex 1.

### 3.5 Estimation Model of Warfarin Dose in Patients with Different Genotypes

Based on the statistical results, we recommend using the following algorithm to predict warfarin maintenance dose for patients with different heart valve replacement genotypes:

Dose (mg/day) = 2.070 + 0.820 × BSA (/100 $m^{2}$) – 0.001 × UA + 0.692 × CV2 – 0.553 × CV3.

CV2: CYP2C9*1/*1/*VKORC1*(GA+GG);

CV3: CYP2C9*1/*3/*VKORC1*AA.

## 4. Discussion

Patients with mechanical heart valve replacement need to have life-time anticoagulation with warfarin [9]. Since warfarin metabolism is affected by the patient’s age, gender, genotype, drugs and other factors, the therapeutic window of warfarin is narrow and serious complications such as bleeding or thrombosis may occur. Therefore, patients need to continually adjust the dose to avoid complications. Warfarin doses taken by patients have been shown to correlate with body surface area and genotyping [10]. Clinical data show that pharmacokinetic genetic algorithms are superior to traditional dosing methods in reducing patient doses with warfarin anticoagulation, and can better predict the proper maintenance dose. The results of this study suggest that independent factors affecting the maintenance dose of warfarin include patient body surface area, genotype, and UA content.

Genotyping analysis can predict warfarin maintenance dose [11]. Therapeutic doses of warfarin can be estimated by genotyping SNPs in patients that can affect warfarin metabolism or sensitivity. Warfarin doses are typically low in patients who are sensitive to warfarin homozygous for common *VKORC1* promoter polymorphisms [5]. Huang Q *et al*. [12] collected preemptive pharmacogenomics (PGx) detection data of 22,918 participants, analyzed the frequency, genotype, and drug genotype of alleles, and predicted the drug response of each participant. Their findings showed that more than 99 percent of participants were advised to reduce their warfarin dose based on genetic factors. Our data showed that the maintenance dose of warfarin anticoagulation in patients undergoing mechanical heart valve replacement was highest in the CYP2C9*1/*1/*VKORC1*(GA+GG) group, followed by CYP2C9*1/*3/*VKORC1*(GA+GG) group and CYP2C9*1/*1/*VKORC1* mutant AA group, and the lowest in the CYP2C9*1/*3/*VKORC1* mutant AA group, which is consistent with a previous study [13]. These data indicate that genotyping can provide an initial dose range for clinical warfarin anticoagulation.

Data from Vandell AG *et al*. [14] showed that the *CYP2C9* and *VKORC1* genotypes were able to identify patients with venous thromboembolism (VTE) who were at increased risk of bleeding due to warfarin anticoagulation. Many warfarin-treated patients develop acute bleeding and the INR in many patients on warfarin exceeded the target for their condition [15]. Gage BF *et al*. [16] conducted a clinical data analysis on 1650 randomized patients taking warfarin and found that the incidence of major bleeding and VTE in the genotype guidance group was significantly lower than that in the clinical guidance group. Mega JL *et al*. [17] reported that *CYP2C9* and *VKORC1* genotypes can predict the risk of early bleeding in patients receiving warfarin anticoagulation, which is independent from clinical risk scores.

On the contrary, there are also studies to show that the warfarin genotype has a limited effect on guiding clinical dosing. Wen MS *et al*. [18] investigated the clinical utility of genotype-guided doses of warfarin and showed that genotype-guided doses did not provide a clear benefit. They concluded that frequent INR monitoring was adequate to effectively control the anticoagulant effect of warfarin. Hao Y *et al*. [19] also suggested that warfarin pharmacogenetic testing according to the algorithm of the International Warfarin Pharmacogenetics Alliance did not improve the anticoagulation results of patients with heart valve replacement in China. This may be because the anticoagulant effect of warfarin is affected by drug metabolism, absorption and other factors. Therefore, accounting for all these influencing factors can provide a theoretical basis for guiding the dose of warfarin and shortening the time to achieve an appropriate maintenance dose. In this study, we mainly investigated the correlation between metabolic indexes and warfarin dose in patients with different genotypes of heart valve replacement.

Age is one of the factors that affect warfarin dose [20]. With aging, the metabolic function of patients changes. It is unknown as to whether changes in liver function, kidney function and UA will affect the metabolism, distribution, absorption and excretion of warfarin. It is unclear whether metabolic markers in patients with different genotypes of heart valve replacement are related to warfarin maintenance dose. Studies have shown that non-genetic factors such as liver function and kidney function are associated with warfarin maintenance dose [21]. Lip GYH *et al*. [22] found that abnormal liver function and abnormal kidney function in warfarin anticoagulation patients were important factors in predicting bleeding risk based on multivariate analysis.

Warfarin is a vitamin K anticoagulant that exerts an anticoagulant effect by inhibiting vitamin K-dependent coagulation proteins [23, 24]. Vitamin K metabolism is mainly affected by liver function; therefore changes in liver function may affect warfarin metabolism. Wang D *et al*. [25] detected the expression of *VKORC1* allele mRNA in human liver, heart, and B lymphocytes. This genotypic effect is selectively observed in the liver, but not in the heart or lymphocytes. Our data suggest that maintenance doses of warfarin in patients undergoing heart valve replacement with different genotypes are not significantly associated with liver function.

Estimated glomerular filtration rate (eGFR) in patients with kidney insufficiency had a significant effect on the warfarin maintenance dose, and a 10 mL/min/1.73 $m^{2}$ increase in eGFR in patients increased the warfarin maintenance dose by 0.6 mg [14]. In patients with severe renal insufficiency, apixaban may be a reasonable alternative to warfarin [26]. Lee WC *et al*. [27] compared baseline eGFR, follow-up eGFR, and changes in eGFR versus baseline eGFR at 2 years between different DOACs and warfarin. The results showed that warfarin was associated with a higher incidence of acute kidney injury (AKI) compared with DOACs over an average observation period of 3.3 ± 0.9 years. There was no difference in decreased kidney function between warfarin and different DOAC groups. On the other hand, warfarin anticoagulation has a detrimental effect on kidney function [28]. Our study suggested that kidney function in patients undergoing heart valve replacement with different genotypes is not significantly associated with maintenance doses of warfarin.

Compared to other oral anticoagulants, warfarin does not exert any UA lowering effect [29]. A previous study [30] showed that the increase of UA in plasma with warfarin administration is probably due to an increase in UA production and those patients may predispose to gout who are on long-term therapy with warfarin. Zhang X *et al*. [31] evaluated the risk of thrombosis from three anticoagulants, including warfarin, and the results showed that abnormal UA metabolism is an independent risk marker for left ventricular thrombosis. Our data suggested that UA levels in patients undergoing heart valve replacement with different genotypes are significantly associated with the maintenance dose of warfarin. This may be due to the fact that the patient’s UA level affects the metabolism and excretion of warfarin, affects the anticoagulant effect of warfarin, and thus affects the dose of warfarin. Therefore, when clinically predicting the maintenance dose of warfarin in patients with different genotypes of heart valve replacement, the maintenance dose of warfarin will be more objectively estimated by referring to the UA content of the patient.

In view of the many factors affecting the compliance of patients with warfarin anticoagulation, and the complications such as bleeding and thrombosis associated with the use of warfarin, in recent years, clinical studies have been conducted to test DOACs as a substitute for warfarin. Clinical data suggest that dabigatran anticoagulation in patients with mechanical heart valves significantly increases thromboembolic and bleeding complications compared with warfarin [3]. Warfarin was not associated with mortality, ischemic stroke, and hemorrhagic stroke events in patients with atrial fibrillation undergoing hemodialysis [32]. Warfarin is associated with improved overall survival in patients receiving cancer-associated VTE therapy compared with low molecular weight heparin [33]. Compared with warfarin, DOACs reduce the incidence of stroke, bleeding, and mortality [21, 34], but cost limits their use in some patients [35]. For these reasons, warfarin remains the anticoagulant of choice for patients undergoing mechanical heart valve replacement. Therefore, it is of great clinical significance to determine the personalized treatment of warfarin in patients, shorten the time of drug dose adjustment, and reduce complications.

This study suggested that clinicians should consider indicators related to the patient’s metabolism while considering the application of drug genetic algorithms to personalize the initial dose of warfarin in Asian patients. Our findings indicated that maintenance doses of warfarin correlate with UA content in patients with different genotypes. Therefore, the comprehensive evaluation of warfarin genotype analysis and metabolic indexes such as UA in patients undergoing mechanical heart valve replacement will provide a theoretical basis for the individualized warfarin anticoagulation of patients. We will continue to study the correlation between the genotype and warfarin dose in patients undergoing heart valve replacement in this region. We hope to establish a more objective mathematical formula through more clinical data, so as to calculate the appropriate warfarin dose based on the patient’s genotype and related clinical indicators, shorten the time to adjust the dose, and reduce complications such as bleeding and thromboembolism.

The present study has several limitations. First, due to the relatively small proportion of patients with genotype CYP2C9*1/*3, and genotypes *CYP2C9* (GA) and *CYP2C9* (GG), the number of patients was small and we combined *CYP2C9* (GA) and *CYP2C9* (GG) for statistical analysis. Due to the limited sample size, the number of CYP2C9*1/*3/*CYP2C9*(GA+GG) groups was too small to accurately reflect the correlation between warfarin dose and UA in patients with different genotypes. We will continue to accumulate larger sample sizes in follow-up studies to further refine the data. Second, we included patients in this study with metabolic-related indicators that were outside the normal range but did not have liver failure or kidney failure. Therefore, if there is liver failure or kidney failure in the clinic, physicians should pay attention to the metabolism and excretion of warfarin, and adjust the dose of warfarin. Third, the mechanism by which UA content in patients with different genotypes of heart valve replacement is significantly associated with the maintenance dose of warfarin is unclear and needs to be further investigated.

## 5. Conclusions

Our study found a significant correlation between UA content and warfarin dose in patients with heart valve replacement genotypes CYP2C9*1/*1/VKORC1(GA+GG), CYP2C9*1/*1/VKORC1AA, CYP2C9*1/*1/VKORC1AA. The accuracy of the maintenance dose of warfarin can be estimated by taking into account the patient’s genotype analysis, age, height, weight, genotype, and UA level, thus shortening the time it takes for patients to achieve a therapeutic dose of warfarin and reduce the potential risk of bleeding or thrombosis.

## Data Availability

The datasets used in this study are available from the corresponding author on reasonable request.

## Supplementary Material

Supplementary material associated with this article can be found, in the online version, at https://doi.org/10.31083/j.rcm2504128.
