# Supplementary material for: Correlation between Metabolic Parameters and Warfarin Dose in Patients with Heart Valve Replacement of Different Genotypes
Source: Rev Cardiovasc Med. 2024 Apr 1;25(4):128. doi: 10.31083/j.rcm2504128 (PMC11264039; doi:10.31083/j.rcm2504128)
Supplement: Supplementary file 1 [file 2153-8174-25-4-128-s1.zip › 2153-8174-25-4-128-s1/Data 2.pdf]

T-Test

Group Statistics

	(sex)	N	Mean	Std. Deviation	Std. Error Mean
(warfarin)	1.00	208	3.0350	.89665	.06217
	.00	135	2.8262	.96973	.08346

Independent Samples Test

		Levene's Test for Equality of Variances		t-test for Equality of Means						
									95% Confidence Interval of the Difference	
		F	Sig.	t	df	Sig. (2-tailed)	Mean Difference	Std. Error Difference	Lower	Upper
(warfarin)	Equal variances assumed	.857	.355	2.040	341	.042	.20881	.10235	.00749	.41012
	Equal variances not assumed			2.006	270.129	.046	.20881	.10407	.00391	.41370

Correlations

Correlations

		(warfarin)	(age)
(warfarin)	Pearson Correlation	1	-.108*
	Sig. (2-tailed)		.046
	N	343	343
(age)	Pearson Correlation	-.108*	1
	Sig. (2-tailed)	.046	
	N	343	343

*. Correlation is significant at the 0.05 level (2-tailed).

```
NONPAR CORR
  /VARIABLES=VAR00001 VAR00003
  /PRINT=SPEARMAN TWOTAIL NOSIG
  /MISSING=PAIRWISE.
```

Nonparametric Correlations

Correlations

			(warfarin)	(age)
Spearman's rho	(warfarin)	Correlation Coefficient	1.000	-.154**
		Sig. (2-tailed)	.	.004
		N	343	343
	(age)	Correlation Coefficient	-.154**	1.000
		Sig. (2-tailed)	.004	.
		N	343	343

**. Correlation is significant at the 0.01 level (2-tailed).

```
CORRELATIONS
  /VARIABLES=VAR00001 VAR00002
  /PRINT=TWOTAIL NOSIG
  /MISSING=PAIRWISE.
```

Correlations

Correlations

		(warfarin)	BSA
(warfarin)	Pearson Correlation	1	.177**
	Sig. (2-tailed)		.001
	N	343	343
BSA	Pearson Correlation	.177**	1
	Sig. (2-tailed)	.001	
	N	343	343

**. Correlation is significant at the 0.01 level (2-tailed).

```
NONPAR CORR
  /VARIABLES=VAR00001 VAR00002
  /PRINT=SPEARMAN TWOTAIL NOSIG
  /MISSING=PAIRWISE.
```

Nonparametric Correlations

Correlations

			(warfarin)	BSA
Spearman's rho	(warfarin)	Correlation Coefficient	1.000	.171**
		Sig. (2-tailed)	.	.002
		N	343	343
	BSA	Correlation Coefficient	.171**	1.000
		Sig. (2-tailed)	.002	.
		N	343	343

**. Correlation is significant at the 0.01 level (2-tailed).

```
CORRELATIONS
  /VARIABLES=VAR00001 VAR00004
  /PRINT=TWOTAIL NOSIG
  /MISSING=PAIRWISE.
```

Correlations

Correlations

		(warfarin)	(ALT)
(warfarin)	Pearson Correlation	1	.029
	Sig. (2-tailed)		.589
	N	343	343
(ALT)	Pearson Correlation	.029	1
	Sig. (2-tailed)	.589	
	N	343	343

```
NONPAR CORR
  /VARIABLES=VAR00001 VAR00004
  /PRINT=SPEARMAN TWOTAIL NOSIG
  /MISSING=PAIRWISE.
```

Nonparametric Correlations

Correlations

			(warfarin)	(ALT)
Spearman's rho	(warfarin)	Correlation Coefficient	1.000	.057
		Sig. (2-tailed)	.	.289
		N	343	343
	(ALT)	Correlation Coefficient	.057	1.000
		Sig. (2-tailed)	.289	.
		N	343	343

```
T-TEST GROUPS=VAR00015(1 0)
  /MISSING=ANALYSIS
  /VARIABLES=y
  /CRITERIA=CI(.95).
```

T-Test

Group Statistics

	(Ethnicity)	N	Mean	Std. Deviation	Std. Error Mean
(warfarin)	1.00	339	2.9554	.93447	.05075
	.00	4	2.7344	.46875	.23438

Independent Samples Test

		Levene's Test for Equality of Variances		t-test for Equality of Means						
									95% Confidence Interval of the Difference	
		F	Sig.	t	df	Sig. (2-tailed)	Mean Difference	Std. Error Difference	Lower	Upper
(warfarin)	Equal variances assumed	1.607	.206	.472	341	.637	.22107	.46843	-.70031	1.14245
	Equal variances not assumed			.922	3.288	.419	.22107	.23981	-.50566	.94780

```
CORRELATIONS
  /VARIABLES=VAR00001 VAR00005
  /PRINT=TWOTAIL NOSIG
  /MISSING=PAIRWISE.
```

Correlations

Correlations

		(warfarin)	(AST)
(warfarin)	Pearson Correlation	1	-.030
	Sig. (2-tailed)		.577
	N	343	343
(AST)	Pearson Correlation	-.030	1
	Sig. (2-tailed)	.577	
	N	343	343

```
NONPAR CORR
  /VARIABLES=VAR00001 VAR00005
  /PRINT=SPEARMAN TWOTAIL NOSIG
  /MISSING=PAIRWISE.
```

Nonparametric Correlations

Correlations

			(warfarin)	(AST)
Spearman's rho	(warfarin)	Correlation Coefficient	1.000	-.020
		Sig. (2-tailed)	.	.708
		N	343	343
	(AST)	Correlation Coefficient	-.020	1.000
		Sig. (2-tailed)	.708	.
		N	343	343

```
CORRELATIONS
  /VARIABLES=VAR00001 VAR00013
  /PRINT=TWOTAIL NOSIG
  /MISSING=PAIRWISE.
```

Correlations

Correlations

		VAR00001	VAR00013
VAR00001	Pearson Correlation	1	-.111*
	Sig. (2-tailed)		.040
	N	341	340
VAR00013	Pearson Correlation	-.111*	1
	Sig. (2-tailed)	.040	
	N	340	342

*. Correlation is significant at the 0.05 level (2-tailed).

```
NONPAR CORR
  /VARIABLES=VAR00001 VAR00013
  /PRINT=SPEARMAN TWOTAIL NOSIG
  /MISSING=PAIRWISE.
```

Nonparametric Correlations

Correlations

			VAR00001	VAR00013
Spearman's rho	VAR00001	Correlation Coefficient	1.000	-.111*
		Sig. (2-tailed)	.	.040
		N	341	340
	VAR00013	Correlation Coefficient	-.111*	1.000
		Sig. (2-tailed)	.040	.
		N	340	[illegible]

```
CORRELATIONS
  /VARIABLES=VAR00001 VAR00008
  /PRINT=TWOTAIL NOSIG
  /MISSING=PAIRWISE.
```

Correlations

Correlations

		(warfarin)	(ALP)
(warfarin)	Pearson Correlation	1	-.072
	Sig. (2-tailed)		.182
	N	343	343
(ALP)	Pearson Correlation	-.072	1
	Sig. (2-tailed)	.182	
	N	343	343

```
NONPAR CORR
  /VARIABLES=VAR00001 VAR00008
  /PRINT=SPEARMAN TWOTAIL NOSIG
  /MISSING=PAIRWISE.
```

Nonparametric Correlations

Correlations

			(warfarin)	(ALP)
Spearman's rho	(warfarin)	Correlation Coefficient	1.000	-.073
		Sig. (2-tailed)	.	.176
		N	343	343
	(ALP)	Correlation Coefficient	-.073	1.000
		Sig. (2-tailed)	.176	.
		N	343	343

```
CORRELATIONS
  /VARIABLES=VAR00001 VAR00009
  /PRINT=TWOTAIL NOSIG
  /MISSING=PAIRWISE.
```

Correlations

Correlations

		(warfarin)	(GGT)
(warfarin)	Pearson Correlation	1	-.024
	Sig. (2-tailed)		.655
	N	343	343
(GGT)	Pearson Correlation	-.024	1
	Sig. (2-tailed)	.655	
	N	343	343

```
NONPAR CORR
  /VARIABLES=VAR00001 VAR00009
  /PRINT=SPEARMAN TWOTAIL NOSIG
  /MISSING=PAIRWISE.
```

Nonparametric Correlations

Correlations

			(warfarin)	(GGT)
Spearman's rho	(warfarin)	Correlation Coefficient	1.000	-.014
		Sig. (2-tailed)	.	.800
		N	343	343
	(GGT)	Correlation Coefficient	-.014	1.000
		Sig. (2-tailed)	.800	.
		N	343	343

```
CORRELATIONS
  /VARIABLES=VAR00001 VAR00010
  /PRINT=TWOTAIL NOSIG
  /MISSING=PAIRWISE.
```

Correlations

Correlations

		(warfarin)	(BUN)
(warfarin)	Pearson Correlation	1	-.007
	Sig. (2-tailed)		.890
	N	343	343
(BUN)	Pearson Correlation	-.007	1
	Sig. (2-tailed)	.890	
	N	343	343

```
NONPAR CORR
  /VARIABLES=VAR00001 VAR00010
  /PRINT=SPEARMAN TWOTAIL NOSIG
  /MISSING=PAIRWISE.
```

Nonparametric Correlations

Correlations

			(warfarin)	(BUN)
Spearman's rho	(warfarin)	Correlation Coefficient	1.000	-.027
		Sig. (2-tailed)	.	.612
		N	343	343
	(BUN)	Correlation Coefficient	-.027	1.000
		Sig. (2-tailed)	.612	.
		N	343	343

```
CORRELATIONS
  /VARIABLES=VAR00001 VAR00017
  /PRINT=TWOTAIL NOSIG
  /MISSING=PAIRWISE.
```

Correlations

Correlations

		VAR00001	VAR00017
VAR00001	Pearson Correlation	1	.081
	Sig. (2-tailed)		.136
	N	343	343
VAR00017	Pearson Correlation	.081	1
	Sig. (2-tailed)	.136	
	N	343	343

```
NONPAR CORR
  /VARIABLES=VAR00001 VAR00017
  /PRINT=SPEARMAN TWOTAIL NOSIG
  /MISSING=PAIRWISE.
```

Nonparametric Correlations

Correlations

			VAR00001	VAR00017
Spearman's rho	VAR00001	Correlation Coefficient	1.000	.100
		Sig. (2-tailed)	.	.064
		N	343	343
	VAR00017	Correlation Coefficient	.100	1.000
		Sig. (2-tailed)	.064	.
		N	343	343

```
CORRELATIONS
  /VARIABLES=VAR00001 VAR00012
  /PRINT=TWOTAIL NOSIG
  /MISSING=PAIRWISE.
```

Correlations

Correlations

		(warfarin)	(UA)
(warfarin)	Pearson Correlation	1	-.111*
	Sig. (2-tailed)		.040
	N	343	343
(UA)	Pearson Correlation	-.111*	1
	Sig. (2-tailed)	.040	
	N	343	343

*. Correlation is significant at the 0.05 level (2-tailed).

```
NONPAR CORR
  /VARIABLES=VAR00001 VAR00012
  /PRINT=SPEARMAN TWOTAIL NOSIG
  /MISSING=PAIRWISE.
```

Nonparametric Correlations

Correlations

			(warfarin)	(UA)
Spearman's rho	(warfarin)	Correlation Coefficient	1.000	-.121*
		Sig. (2-tailed)	.	.025
		N	343	343
	(UA)	Correlation Coefficient	-.121*	1.000
		Sig. (2-tailed)	.025	.
		N	343	343

*. Correlation is significant at the 0.05 level (2-tailed).

```
CORRELATIONS
  /VARIABLES=VAR00001 VAR00011
  /PRINT=TWOTAIL NOSIG
  /MISSING=PAIRWISE.
```

Correlations

Correlations

		VAR00001	VAR00011
VAR00001	Pearson Correlation	1	-.024
	Sig. (2-tailed)		.661
	N	342	342
VAR00011	Pearson Correlation	-.024	1
	Sig. (2-tailed)	.661	
	N	342	343

```
NONPAR CORR
  /VARIABLES=VAR00001 VAR00011
  /PRINT=SPEARMAN TWOTAIL NOSIG
  /MISSING=PAIRWISE.
```

Nonparametric Correlations

Correlations

			VAR00001	VAR00011
Spearman's rho	VAR00001	Correlation Coefficient	1.000	-.011
		Sig. (2-tailed)	.	.837
		N	342	342
	VAR00011	Correlation Coefficient	-.011	1.000
		Sig. (2-tailed)	.837	.
		N	342	343

```
CORRELATIONS
  /VARIABLES=VAR00001 VAR00014
  /PRINT=TWOTAIL NOSIG
  /MISSING=PAIRWISE.
```

Correlations

Correlations

		(warfarin)	(ALB)
(warfarin)	Pearson Correlation	1	.036
	Sig. (2-tailed)		.508
	N	343	343
(ALB)	Pearson Correlation	.036	1
	Sig. (2-tailed)	.508	
	N	343	343

```
NONPAR CORR
  /VARIABLES=VAR00001 VAR00014
  /PRINT=SPEARMAN TWOTAIL NOSIG
  /MISSING=PAIRWISE.
```

Nonparametric Correlations

Correlations

			(warfarin)	(ALB)
Spearman's rho	(warfarin)	Correlation Coefficient	1.000	-.011
		Sig. (2-tailed)	.	.835
		N	343	343
	(ALB)	Correlation Coefficient	-.011	1.000
		Sig. (2-tailed)	.835	.
		N	343	343

```
ONEWAY y BY VAR00017
  /STATISTICS HOMOGENEITY
  /MISSING ANALYSIS.
```

Oneway

Test of Homogeneity of Variances

(warfarin)

Levene Statistic	df1	df2	Sig.
20.465	1	341	.000

ANOVA

(warfarin)

	Sum of Squares	df	Mean Square	F	Sig.
Between Groups	5.208	1	5.208	6.107	.014
Within Groups	290.796	341	.853		
Total	296.004	342			

```
ONEWAY y BY VAR00018
  /STATISTICS HOMOGENEITY
  /MISSING ANALYSIS.
```

Oneway

Test of Homogeneity of Variances

(warfarin)

Levene Statistic	df1	df2	Sig.
16.937	1	341	.000

ANOVA

(warfarin)

	Sum of Squares	df	Mean Square	F	Sig.
Between Groups	27.270	1	27.270	34.603	.000
Within Groups	268.734	341	.788		
Total	296.004	342			

```
ONEWAY y BY VAR00019
  /STATISTICS HOMOGENEITY
  /MISSING ANALYSIS.
```

Oneway

Test of Homogeneity of Variances

(warfarin)

Levene Statistic	df1	df2	Sig.
.046	1	341	.830

ANOVA

(warfarin)

	Sum of Squares	df	Mean Square	F	Sig.
Between Groups	14.069	1	14.069	17.016	.000
Within Groups	281.935	341	.827		
Total	296.004	342			

```
ONEWAY y BY VAR00020
  /STATISTICS HOMOGENEITY
  /MISSING ANALYSIS.
```

Oneway

Test of Homogeneity of Variances

(warfarin)

Levene Statistic	df1	df2	Sig.
2.328	1	341	.128

ANOVA

(warfarin)

	Sum of Squares	df	Mean Square	F	Sig.
Between Groups	.060	1	.060	.070	.792
Within Groups	295.943	341	.868		
Total	296.004	342			
