# Supplementary material for: Correlation between Metabolic Parameters and Warfarin Dose in Patients with Heart Valve Replacement of Different Genotypes
Source: Rev Cardiovasc Med. 2024 Apr 1;25(4):128. doi: 10.31083/j.rcm2504128 (PMC11264039; doi:10.31083/j.rcm2504128)
Supplement: Supplementary file 1 [file 2153-8174-25-4-128-s1.zip › 2153-8174-25-4-128-s1/Data 3.pdf]

Variables Entered/Removed[a]

Model	Variables Entered	Variables Removed	Method
1	13GAGG, (sex), 13AA, (UA), 11GAGG, (age), BSA[b]	.	Enter

a. Dependent Variable: (warfarin)

b. Tolerance = .000 limit reached.

Model Summary

Model	R	R Square	Adjusted R Square	Std. Error of the Estimate
1	.420[a]	.176	.159	.85305

a. Predictors: (Constant), 13GAGG, (sex), 13AA, (UA), 11GAGG, (age), BSA

ANOVA[a]

Model		Sum of Squares	df	Mean Square	F	Sig.
1	Regression	52.228	7	7.461	10.253	.000[b]
	Residual	243.776	335	.728		
	Total	296.004	342			

a. Dependent Variable: (warfarin)

b. Predictors: (Constant), 13GAGG, (sex), 13AA, (UA), 11GAGG, (age), BSA

Coefficients[a]

Model		Unstandardized Coefficients		Standardized Coefficients	t	Sig.
		B	Std. Error	Beta		
1	(Constant)	2.070	.597		3.468	.001
	BSA	.820	.312	.160	2.625	.009
	(age)	-.007	.004	-.079	-1.578	.115
	(UA)	-.001	.001	-.117	-2.339	.020
	(sex)	.053	.117	.028	.454	.650
	11GAGG	.692	.121	.289	5.733	.000
	13AA	-.553	.171	-.163	-3.236	.001
	13GAGG	.163	.387	.021	.422	.673

[illegible]

ANOVA[a]

Model		Sum of Squares	df	Mean Square	F	Sig.
1	Regression	52.228	7	7.461	10.253	.000[b]
	Residual	243.776	335	.728		
	Total	296.004	342			

a. Dependent Variable: （warfarin）

b. Predictors: (Constant), 13GAGG, （sex）, 13AA, (UA), 11GAGG, (age), BSA

Coefficients[a]

Model		Unstandardized Coefficients		Standardized Coefficients		
		B	Std. Error	Beta	t	Sig.
1	(Constant)	2.070	.597		3.468	.001
	BSA	.820	.312	.160	2.625	.009
	(age)	-.007	.004	-.079	-1.578	.115
	(UA)	-.001	.001	-.117	-2.339	.020
	（sex）	.053	.117	.028	.454	.650
	11GAGG	.692	.121	.289	5.733	.000
	13AA	-.553	.171	-.163	-3.236	.001
	13GAGG	.163	.387	.021	.422	.673

a. Dependent Variable: （warfarin）

Excluded Variables[a]

Model		Beta In	t	Sig.	Partial Correlation	Collinearity Statistics Tolerance
1	11AA	.[b]	.	.	.	.000

a. Dependent Variable: （warfarin）

b. Predictors in the Model: (Constant), 13GAGG, （sex）, 13AA, (UA), 11GAGG, (age), BSA
